# Protective effects of tiotropium alone or combined with budesonide against cadmium inhalation induced acute neutrophilic pulmonary inflammation in rats

**DOI:** 10.1371/journal.pone.0193610

**Published:** 2018-02-28

**Authors:** Shiwei Zhao, Qi Yang, Zhixi Yu, You Lv, Jianming Zhi, Pascal Gustin, Wenhui Zhang

**Affiliations:** 1 Shanghai Jiao Tong University School of Medicine, Shanghai, China; 2 Department of Anatomy and Physiology, Shanghai Jiao Tong University School of Medicine, Shanghai, China; 3 Department for Functional Sciences, Faculty of Veterinary Medicine, University of Liège, Liège, Belgium; Centre National de la Recherche Scientifique, FRANCE

## Abstract

As a potent bronchodilator, the anti-inflammatory effects of tiotropium and its interaction with budesonide against cadmium-induced acute pulmonary inflammation were investigated. Compared to values obtained in rats exposed to cadmium, cytological analysis indicated a significant decrease of total cell and neutrophil counts and protein concentration in bronchoalveolar lavage fluid (BALF) in rats pretreated with tiotropium (70μg/15ml or 350μg/15ml). Zymographic tests showed a decrease of MMP-2 activity in BALF in rats pretreated only with high concentration of tiotropium. Histological examination revealed a significant decrease of the severity and extent of inflammatory lung injuries in rats pretreated with both tested concentrations of tiotropium. Though tiotropium (70μg/15ml) or budesonide (250μg/15ml) could not reduce cadmium-induced bronchial hyper-responsiveness, their combination significantly decreased bronchial contractile response to methacholine. These two drugs separately decreased the neutrophil number and protein concentration in BALF but no significant interaction was observed when both drugs were combined. Although no inhibitory effects on MMP-2 and MMP-9 was observed in rats pretreated with budesonide alone, the combination with the ineffective dose of tiotropium induced a significant reduction on these parameters. The inhibitory effect of tiotropium on lung injuries was not influenced by budesonide which alone induced a limited action on the severity and extent of inflammatory sites. Our findings show that tiotropium exerts anti-inflammatory effects on cadmium-induced acute neutrophilic pulmonary inflammation. The combination of tiotropium with budesonide inhibits cadmium-induced inflammatory injuries with a synergistic interaction on MMP-2 and MMP-9 activity and airway hyper-responsiveness.

## Introduction

Chronic Obstructive Pulmonary Disease (COPD) is characterized by persistent airflow restriction, resulting a progressive irreversible decline in lung function [1]. Lung function and life quality become dramatically impaired in case of acute exacerbations of COPD (AECOPD) which become the main cause of high mortality and morbidity [2]. According to clinical evidence, neutrophil recruitment is highly increased accompanied with bronchoconstriction during COPD exacerbations [3]. Thus, seeking out the effective treatment to suppress neutrophilic infiltration will be helpful to control AECOPD.

According to GOLD, bronchodilators are recommended as the first line therapy in COPD [1]. These coumpounds can also exert anti-inflammatory properties. The anti-inflammatory effect of long-acting muscarinic antagonists (LAMAs) has been mentioned by several studies *in vitro* and *in vivo* [4–6]. Tiotropium has been reported to have an anti-inflammatory activity on cigarette smoke induced pulmonary inflammation in mice [4] and can inhibit cytokines production from structural and inflammatory cells [5–6]. However, whether tiotropium could reduce the severity and frequency of AECOPD by inhibiting neutrophilic infiltration remains to be clarified.

Inhaled corticosteroids (ICSs) are recommended to reduce the inflammation and control symptoms of AECOPD by GOLD [1], but their therapeutic efficacy against inflammatory responses in patients with COPD is limited [7] and the risk of pneumonia may be increased due to the long-term use of ICS [8]. It has been demonstrated that the combination of an ICS with an anticholinergic agent can improve COPD patients' lung function, clinical symptoms, exercise tolerance and life quality [9]. In conscious guinea pigs, co-administration of budesonide with tiotropium exerted a significant protection against methacholine-induced bronchoconstriction [10]. In contrast, some studies mentioned no additional efficacy to reduce the incidence of AECOPD when tiotropium was added to ICS/long-acting β_2_-adrenergic agonist (LABA) [11]. It needs to clarify whether these combinations, especially LAMAs, could restore ICS insensitivity to well control the inflammatory responses and thereby reduce exacerbation rates of COPD.

Cigarette smoking is the most important risk factor while air pollution and occupational exposure are also recognized as risk factors for COPD. Cadmium (Cd) consists one of tobacco components and can be inhaled by active or passive route through cigarette smoke. Several studies have mentioned that Cd could impair lung function and induce lung diseases involving neutrophilic pulmonary inflammation [12,13]. In our previous study, an acute exposure to Cd has been found to induce bronchoconstriction and neutrophilic infiltration associated with increased MMP-2 and MMP-9 activity which mimic some main features of AECOPD [14].

Matrix metalloproteinases (MMPs), especially MMP-2 and MMP-9 are considered as markers of acute lung inflammation. Evidence has highlighted that inhibition of MMP-2 and MMP-9 provides a protective effect against acute lung injuries [15–17]. The effect of ICS on MMPs activity seems controversial but it has been suggested that the low potency of ICS to decrease MMPs activity may be partially responsible of their lack of efficacy [18–21]. Some studies have revealed the inhibitory effect of anticholinergics on MMP-2 and MMP-9 activity [22–24]. Interactions between these compounds at this level is not documented.

By using the model of acute pulmonary inflammation induced by a single inhalation of Cd in rats, the aim of this study was to investigate whether tiotropium could exert its anti-inflammatory effect and restore budesonide insensitivity against Cd-induced acute neutrophilic inflammation. We also examined whether the expected protective effects were associated with a modulation of MMP-2 and MMP-9 activity in the present model.

## Materials and methods

### Ethics statement

Male Sprague–Dawley rats (n = 68) weighing 200-250g were obtained from Shanghai SLAC Laboratory Animal Co. Ltd. They were housed in the animal facilities of the laboratory for at least 24 h before being exposed to drugs and Cd and had free access to water and food. All experimental protocols were approved by the Experimental Animal Care and Use Committee of Shanghai Jiao Tong University School of Medicine (SYXK(沪)2003-0026).

### Drugs and solution concentration selection

Cadmium chloride and budesonide were purchased from Sigma (USA). Tiotropium was obtained from Trc (Canada). Cadmium chloride was prepared in saline to get a 0.1% solution. Tiotropium and budesonide were prepared in vehicle (0.9% NaCl solution containing 0.1% DMSO). Methacholine (MCh) was prepared to obtain 6 different concentration (10^−7^、3×10^−7^、10^−6^、3×10^−6^、10^−5^、3×10^-5^mol/L). For ethical reasons, a moderate concentration of cadmium (0.1% CdCl_2_) was used in the present study to alleviate the suffering of animals and to reduce the incidence rate of acute lung hemorrhage as well as the mortality of animals. During the experimental protocol, animals were euthanized if dyspnea, anorexia or abnormal behavior occurs. No animals have been excluded in the present study. Two different concentrations of tiotropium (70μg/15ml or 350μg/15ml of nebulized solution) have been determined during preliminary assays showing the bronchodilating effect and anti-inflammatory properties of this agent. The aim of the present study being to investigate whether tiotropium can interact with glucocorticoid to better control Cd-induced acute neutrophilic pulmonary inflammation, a low concentration of budesonide (250μg/15ml of nebulized solution) demonstrating a very limited anti-inflammatory effect has been selected during preliminary assays.

### Study design and experimental protocols

Animals were placed in an exposure chamber (length × width × height: 50 cm × 50 cm × 37 cm) where they were exposed to drugs and Cd. An ultrasonic nebulizer (Yuwell, 402AI, China) was used to give an aerosol output. Animals were first pretreated with tiotropium (70μg/15ml or 350μg/15ml) or budesonide (250μg/15ml) 30 min before being exposed to 0.9%NaCl (n = 6 per group) or 0.1% CdCl_2_ for 60 min (n = 6 per group). A combination of tiotropium (70μg/15ml) with budesonide (250μg/15ml) was administered 30 min before saline or Cd inhalation (n = 6 per group).

A Cd-exposed group (n = 15) was designed in which animals were exposed to vehicle solution followed by an inhalation of 0.1% CdCl_2_ for 60 min while a NaCl group (n = 5) underwent a vehicle solution nebulization 30 min before being exposed to 0.9% NaCl for 60 min.

The rats were sacrificed 24 h after the exposure by a lethal intraperitoneal injection of 50mg/kg pentobarbital. Bronchoalveolar lavage (BALF) was performed by flushing 8ml of saline two times successively in the right lung through a cannula located in the main bronchus. The liquid was then collected for subsequent BALF analysis, whereas the left lung was fixed for histological examination. Meanwhile the inferior part of the trachea and the left main bronchus was obtained for determination of airway responsiveness.

### Bronchoalveolar lavage fluid analysis

#### Cytological analysis

BALF was centrifuged and supernatant was kept at −80°C for further analysis. A total cell count was performed using Invitrogen Countess™ (Invitrogen, C10227, Korea). 150 μl of BALF was used for differential cell count by cytospin centrifugation (Thermo, Shandon Cytospin 4, UK) and Giemsa staining.

#### Determination of BALF matrix metalloproteases activity

The activity of MMP-2 and MMP-9 was detected by gelatin zymography as previously described [14]. After staining with Coomassie blue and discoloration, MMP activity appeared as unstained zones against a blue background. Quantification of MMP activity was performed by using Image J (Image Processing and Analysis in Java). Results were expressed in average arbitrary units (AU) corresponding to pixel density × mm^2^ for the bands of proteolysis which were normalized by the result of a known amount of standard (human pro- enzyme MMP-9, human pro-enzyme MMP-2, oncogene, San Diego, CA, USA).

#### Determination of protein content in BALF

The protein concentration in BALF was measured by using BCA protein assay kit (Thermo Scientific, Pierce™ BCA Protein Assay Kit, USA).The procedure was performed according to the manufacturer's instruction.

#### Detection of cytokines

ELISA was used to analyze the levels of IL-1β and TNF-α in BALF according to the manufacturer's instruction (Shanghai Enzyme-linked Biotechnology Co., Ltd., China).

### Lung histological examination

After fixation, lungs were embedded in paraffin and 2–3 μm transversal slices were cut in the medial lung portion and stained with hematoxylin-eosin coloration. The lung fields (8 per rat) were randomly selected and care was taken to avoid regions containing pleura or large bronchi.

The extent and severity of lung-tissue inflammation were scored by semi-quantitation in a blinded examination as previously described [14].

### Measurement of bronchial contractile response to methacholine (MCh)

The inferior part of the trachea and the left main bronchus were removed immediately after euthanasia. The same bronchial segment about 2 rings was collected and put into ice-cold Kreb's solution.

Tissue was then suspended in 10 ml Kreb's solution, which was maintained at 37°C and gassed with 95% O_2_ and 5% CO_2_, under isotonic tension of 0.7 g. Tissues were allowed to equilibrate for 1 h, after which the Kreb's solution was changed.

Concentration-effect curves were obtained by exposing to the preparation to cumulative increasing concentrations of MCh. The contractile response was measured by using an isometric force transducer (Kent Scientific, Torrington, CT, USA) which was connected to a computerized data acquisition system (PowerLab/8SP, ADInstruments, Castle Hill, NSW, Australia) and recorded on a PC using Chart 5.0 software. The maximum contractile tension (E_max_) was calculated by Scott ratio method.

### Statistical analysis of results

All data were presented as mean ± SEM, and were analyzed by using GraphPad Prism (GraphPad Software, San Diego, CA). One-way analysis of variance (ANOVA) was performed to analyze the differences between groups followed by a post-test using the Student–Newman–Keuls test for comparison between groups. The P < 0.05 was considered as statistically significant.

## Results

### Bronchial contractile response to methacholine

**Fig 1:**
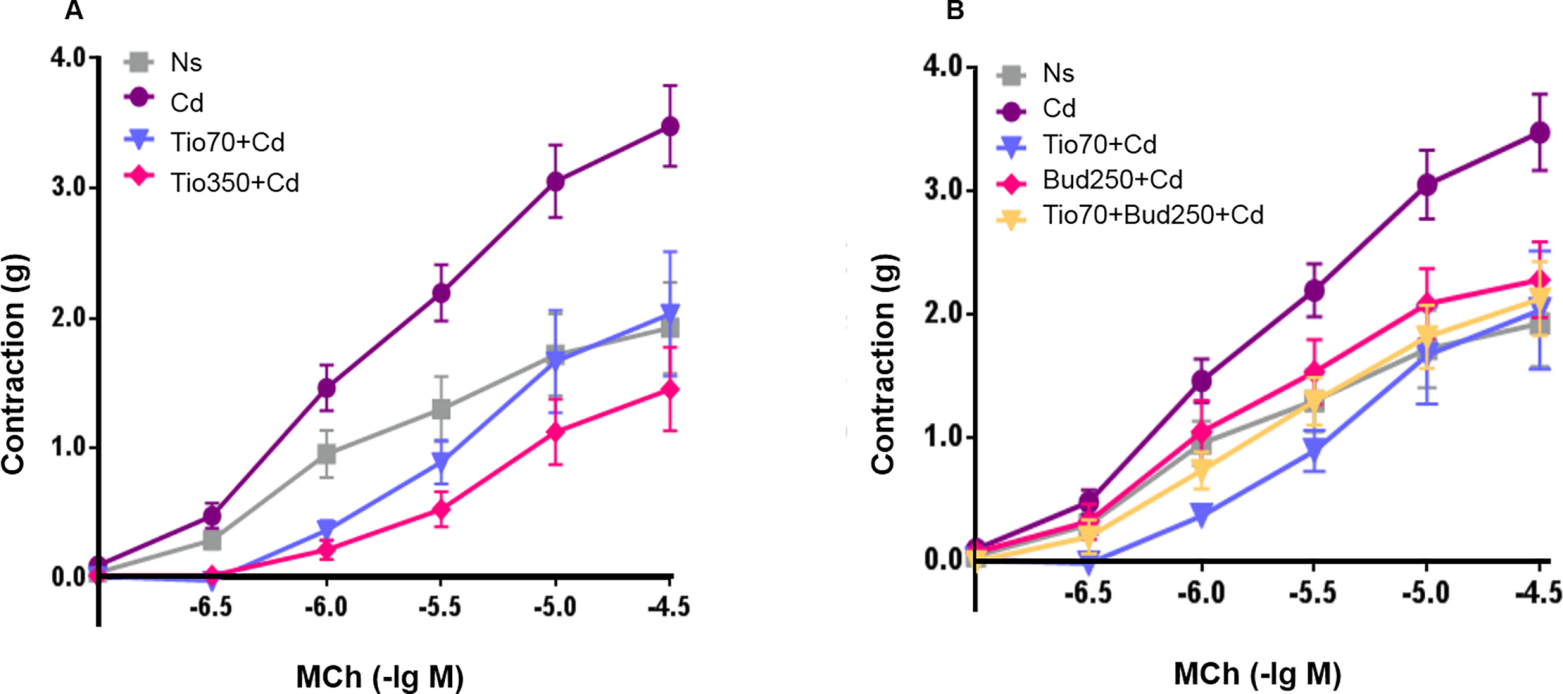
MCh-induced bronchial contraction measured *in vitro* by using the bronchial rings isolated from different experimental groups. Concentration-effect curves were obtained by exposing to the cumulative increasing concentrations of MCh. Data are expressed as the mean ± SEM (n≥5/group). Ns: 0.9% NaCl; Cd: 0.1% CdCl_2_; Tio 70 + Cd, Tio 350 + Cd: animals pretreated with tiotropium 70 μg/15ml or 350 μg/15ml respectively; Bud 250 + Cd: animals pretreated with budesonide 250 μg/15ml; Bud 250 + Tio 70 + Cd: animals pretreated with budesonide 250 μg/15ml and tiotropium 70 μg/15ml followed by cadmium exposure.

Compared with NaCl group, the response of the rat airway smooth muscle to MCh was markedly increased after a single inhalation of Cd (Fig 1A), with mean E_max_ increasing from 2.03±0.38g to 4.08±0.42g (P<0.05). Neither tiotropium nor budesonide exerted any significant influence on airway smooth muscle response on MCh in healthy rats. Compared with Cd group, the pre-administration of tiotropiumat (350 μg/15ml) induced a marked reduction of the response to MCh with a significant decrease of E_max_ value 1.97±0.44g (P<0.05). However, only a slight but not significant decline of the mean E_max_ value (3.37±0.88g) was obtained in rats pretreated with the low concentration of tiotropium (70 μg/15ml).

Budesonide 250 μg/15ml tended to reduce the bronchial contractile response to MCh with the mean E_max_ decreasing to 2.60±0.33g (Fig 1B). This response was significantly enhanced when combined with the low concentration of tiotropium. Indeed, in rats pretreated with both tiotropium (70 μg/15ml) and budesonide (250μg/15ml), a significant reduction of the mean E_max_ value 2.19±0.39g was detected when compared to the data obtained in Cd group (P<0.05).

### Differential cell counts in BALF

**Fig 2:**
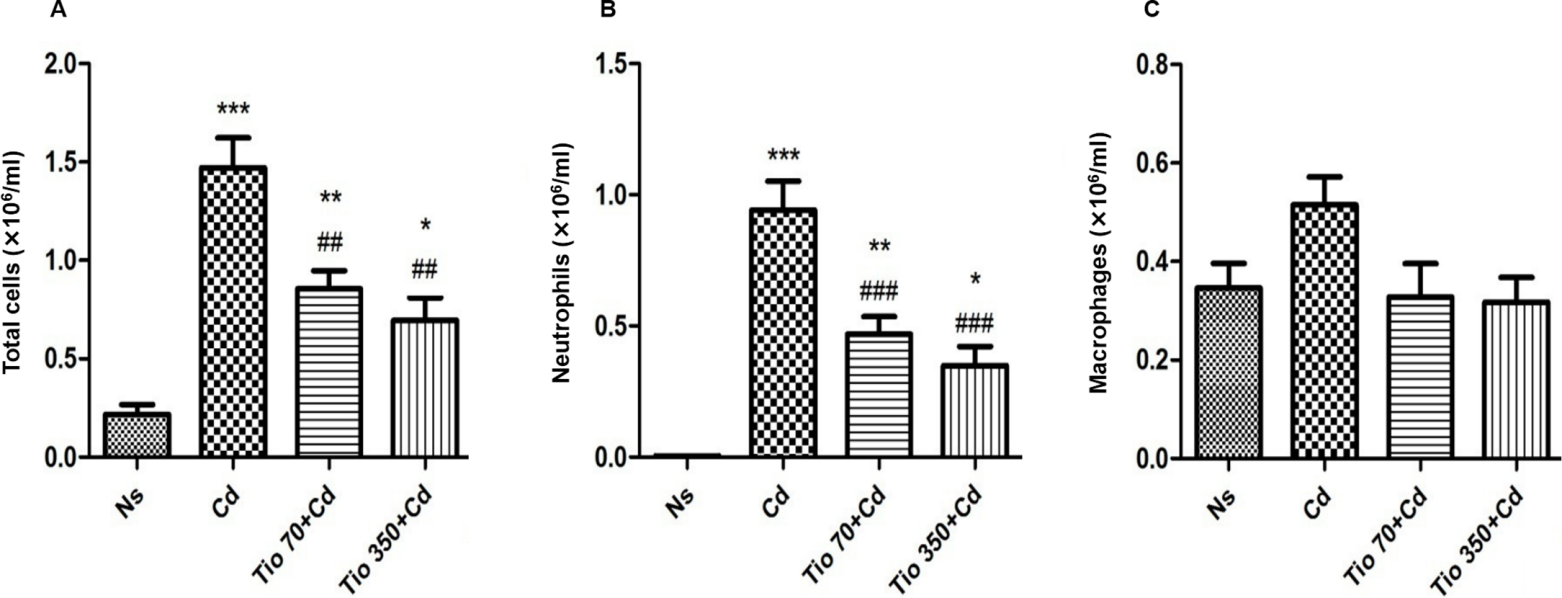
**Effects of increasing concentrations of tiotropium on the number of total cells (A), neutrophils (B) and macrophages (C) in bronchoalveolar lavage fluid in rats exposed to Cd.** For abbreviation meaning, see Fig 1 legend. Data are expressed as the mean ± SEM (n≥5/group). * Indicates a significant difference in comparison with Ns group (* P<0.05, ** P<0.01, *** P<0.001); # indicates a significant difference in comparison with Cd group (# P<0.05, ## P<0.01, ### P<0.001).

Compared with NaCl group, Cd induced a significant increase in total cell number in BALF. This effect was mainly attributed to the marked raise on neutrophil count (Fig 2A and 2B). No significant change of macrophage number in BALF was detected in rats exposed to Cd (Fig 2C). Pretreatment of healthy rats with tiotropium and/or budesonide had no effect on BALF cell counts as compared to NaCl group.

The pretreatment with tiotropium at two different concentrations induced a significant decrease in total cell and neutrophil counts (Fig 2A and 2B). The macrophage number in BALF was not affected by tiotropium (Fig 2C).

**Fig 3:**
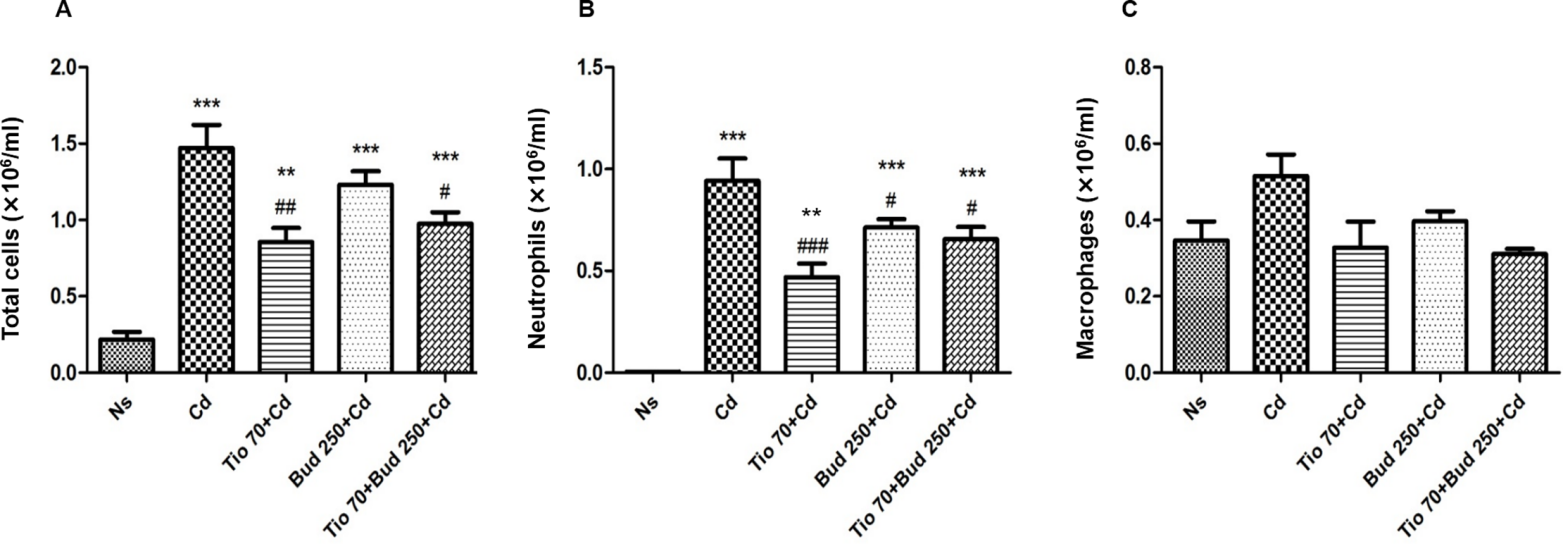
**Effects of tiotropium in combination with budesonide on the number of total cells (A), neutrophils (B) and macrophages (C) in bronchoalveolar lavage fluid in rats exposed to Cd.** For abbreviation meaning, see Fig 1 legend. Data are expressed as the mean ± SEM (n≥5/group). * Indicates a significant difference in comparison with Ns group (* P<0.05, ** P<0.01, *** P<0.001); # indicates a significant difference in comparison with Cd group (# P<0.05, ## P<0.01, ### P<0.001).

Compared with Cd group, budesonide (250 μg/15ml) alone only elicited a more limited but significant decrease in neutrophil number in BALF (Fig 3B). When combined with tiotropium, no interaction was noted on total cell and neutrophil counts in BALF (Fig 3A and 3B). Budesonide alone and combined with the low concentration of tiotropium exerted no effect on macrophage count (Fig 3C).

### Cytokine and total protein level in BALF

**Fig 4:**
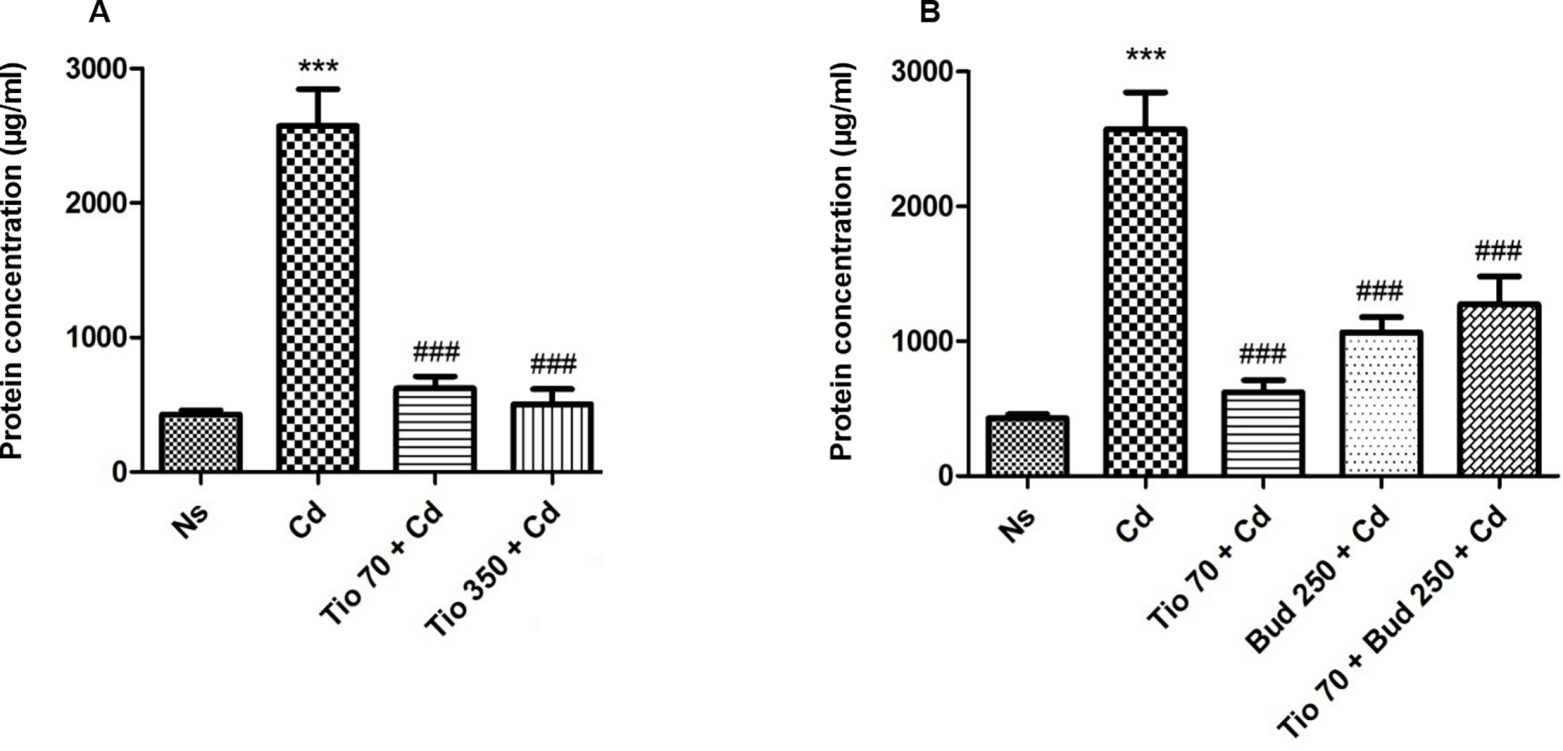
**Protein concentration in bronchoalveolar lavage fluid in rats pretreated with tiotropium alone (A) or combined with budesonide (B).** The protein concentration in BALF was measured by using BCA protein assay kit. For abbreviation meaning, see Fig 1 legend. Data are expressed as the mean ± SEM (n≥5/group). * Indicates a significant difference in comparison with Ns group (* P<0.05, ** P<0.01, *** P<0.001); # indicates a significant difference in comparison with Cd group (# P<0.05, ## P<0.01, ### P<0.001).

Occurrence of edema was suggested by a significant increase in protein concentration in BALF observed in rats exposed to Cd (Fig 4A). No significant change of protein concentration in BALF was detected in healthy rats exposed to both drugs. The pretreatment with the two different concentrations of tiotropium induced a marked reduction in protein concentration. A prominent decrease of protein concentration was also detected in rats pretreated with budesonide (250μg/15ml) alone (Fig 4B). No significant interaction was observed in rats underwent a combined administration of both drugs with a slight loss of efficiency of the combination (Fig 4B).

**Fig 5:**
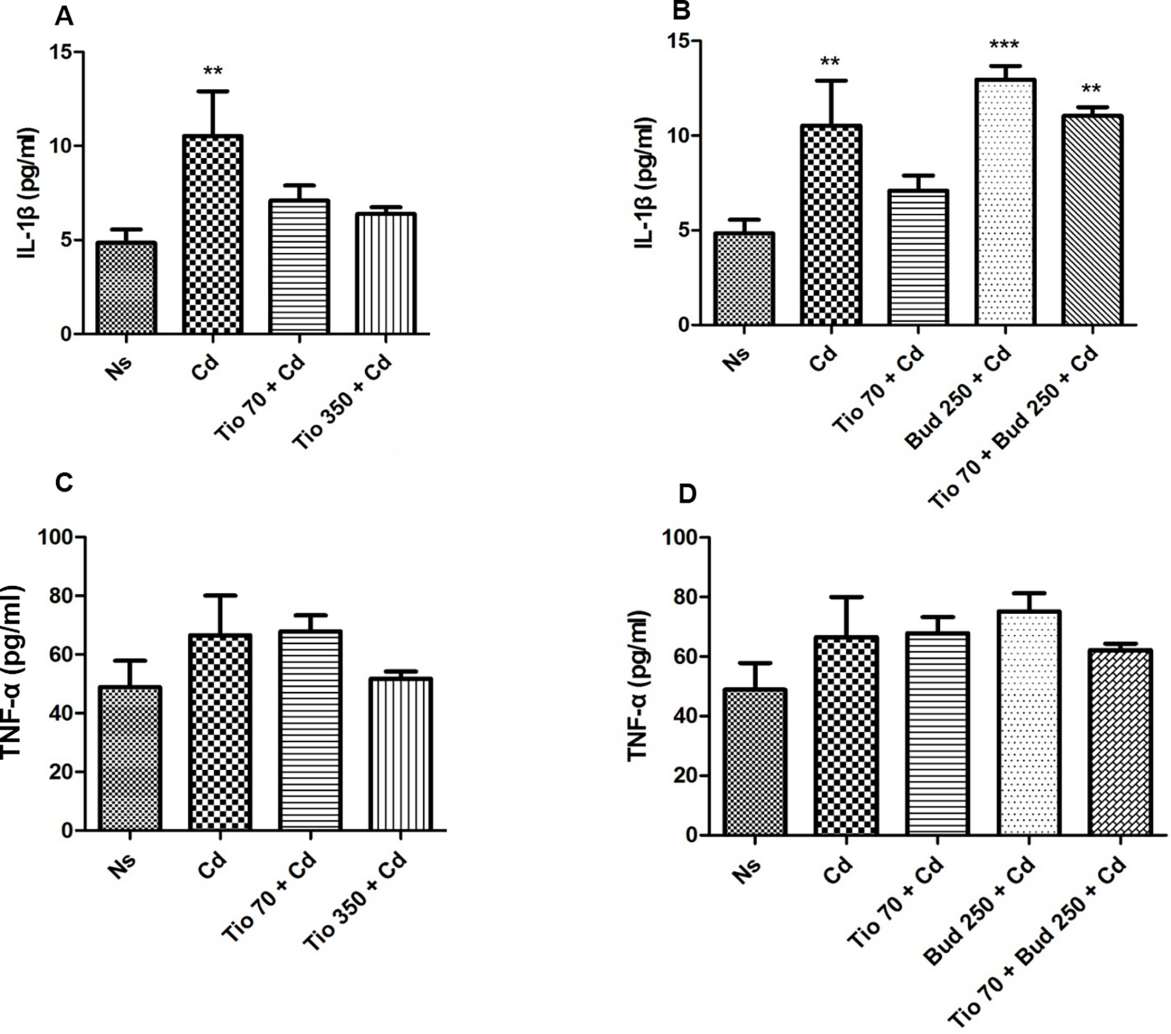
**IL-1β and TNF-α concentrations in bronchoalveolar lavage fluid in rats pretreated with tiotropium alone (A, C) or in combination with budesonide (B, D).** ELISA was used to analyze the concentrations of IL-1β and TNF-α in BALF. For abbreviation meaning, see Fig 1 legend. Data are expressed as the mean ± SEM (n≥5/group). * Indicates a significant difference in comparison with Ns group (* P<0.05, ** P<0.01, *** P<0.001).

A significant increase in IL-1β in BALF was found after a single inhalation of Cd (Fig 5A), while no change of TNF-α concentration in BALF was detected in Cd group (Fig 5C). Tiotropium and/or budesonide had no effect on IL-1β and TNF-α level in healthy rats. A slight decrease of IL-1β concentration in BALF was observed in rats pretreated with two different concentrations of tiotropium (Fig 5A). The effect of Cd became no longer significant suggesting a protective role of tiotropium on this parameter. Budesonide (250μg/15ml) alone or in combination with tiotropium (70 μg/15ml) had no effect on Cd-induced increase of IL-1β level (Fig 5B).

### MMP-2 and MMP-9 gelatinolytic activity in BALF

**Fig 6:**
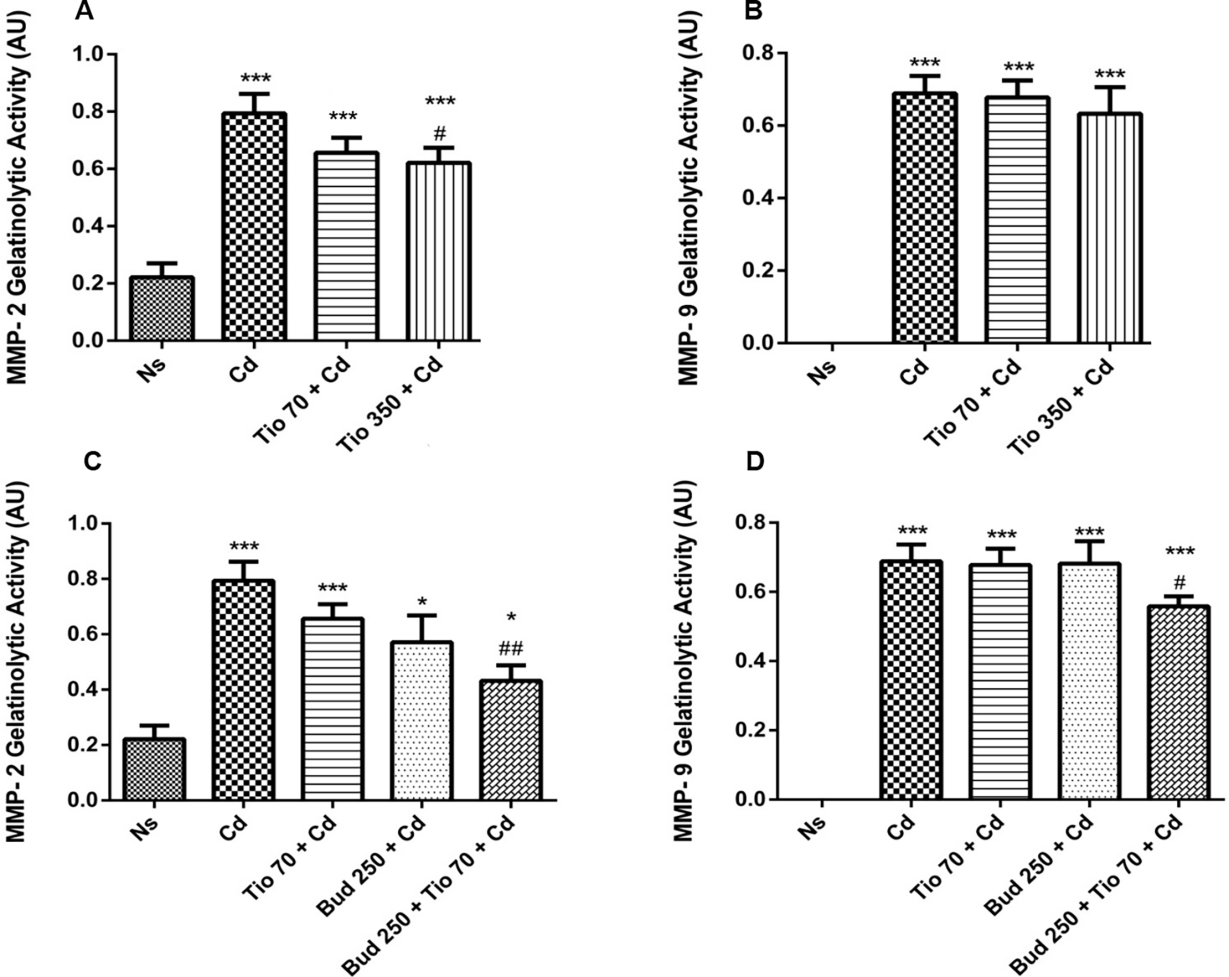
**Effects of tiotropium alone or in combination with budesonide on Cd-induced increase of MMP-2 (A, C) and MMP-9 (B, D) activity in BALF.** Gelatin zymography was used to measure MMP-2 and MMP-9 activity in BALF. For abbreviation meaning, see Fig 1 legend. Data are expressed as the mean ± SEM (n≥5/group). * indicates a significant difference in comparison with Ns group (* P<0.05, *** P<0.001); # indicates a significant difference in comparison with Cd group (# P<0.05, ## P<0.01).

MMP-2 and MMP-9 activities in BALF were significantly increased after a single inhalation of Cd (Fig 6A and 6B). No significant change of MMP-2 and MMP-9 was found in healthy rats exposed to tiotropium and/or budesonide. Tiotropium had no effect on MMP-9 activity, while the high concentration of tiotropium (350 μg/15ml) induced a significant decrease of MMP-2 activity in BALF (Fig 6A and 6B). The administration of budesonide alone failed to reduce Cd-induced increase of both MMP-2 and MMP-9 activity. Compared with Cd group, a significant inhibition on MMP-2 and MMP-9 activity in BALF was found in rats pretreated with budesonide combined with the low concentration of tiotropium indicating a significant interaction between both drugs (Fig 6C and 6D).

### Histological injuries and lung histomorphometry

**Fig 7:**
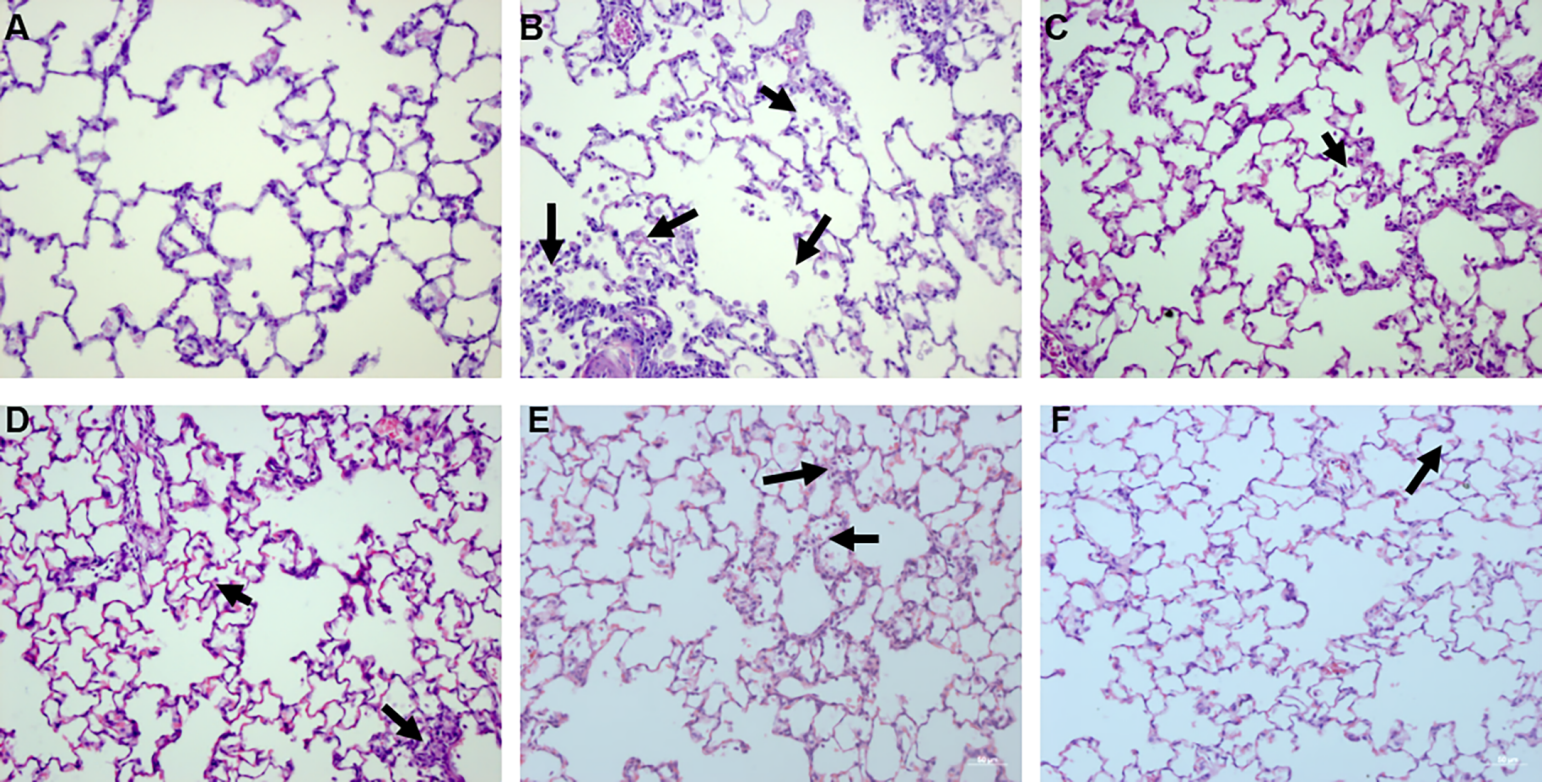
Effect of tiotropium and/or budesonide on the histological injuries induced by Cd inhalation. All sections were stained with hematoxylin-eosin and shown at ×200. Arrows indicate inflammatory cell infiltration into alveoli. A representative lung tissue of different groups is shown (n≥5/group). A: NaCl group, B: CdCl_2_ group, C: Tio 70 + Cd group, D: Tio 350 + Cd group, E: Bud 250 + Cd group, F: Bud 250 + Tio 70 + Cd group.

**Fig 8:**
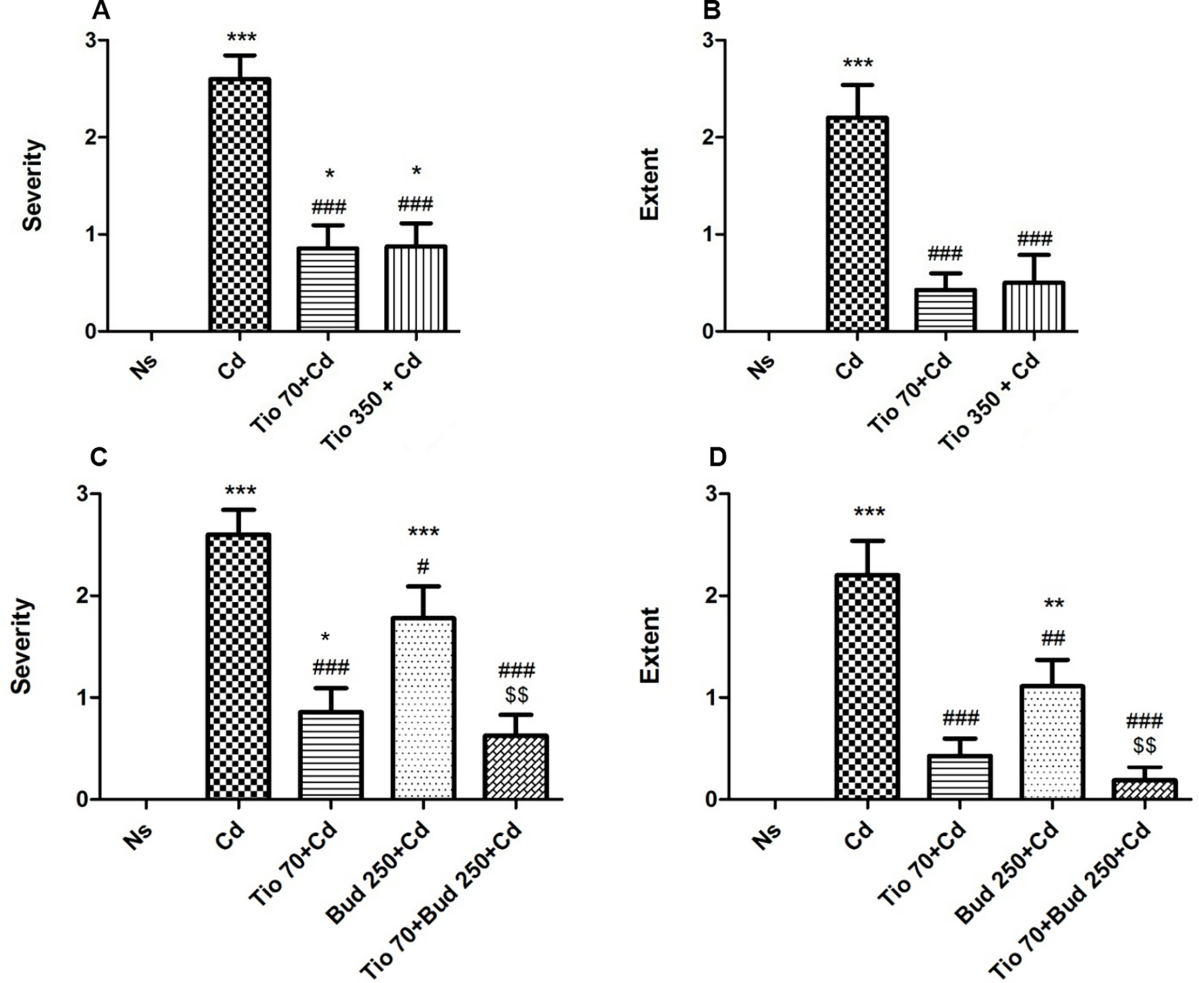
**Inflammatory scores attributed to the severity (A, C) and extent (B, D) of histological injuries.** For abbreviation meaning, see Fig 1 legend. Data are expressed as the mean ± SEM (n≥5/group). * indicates a significant difference in comparison with Ns group (* P<0.05, *** P<0.001); # indicates a significant difference in comparison with Cd group (### P<0.001). $ indicates a significant difference in comparison with Bud 250 + Cd group ($ $ P<0.01).

In NaCl group, the lung architecture was preserved, with unimpaired parenchyma and absence of inflammatory infiltration (Fig 7A). Similar histomorphological characteristics were observed in healthy rats pretreated with tiotropium and/or budesonide. Cd inhalation induced marked pathological changes in the lung tissue. Neutrophil and macrophage infiltration was observed in the alveoli. In the peri-bronchiolar regions and parenchyma, inflammatory infiltration was found with focal congestion and hemorrhage (Fig 7B). The semi-quantitative analysis allowed the measurement of the severity and extent of inflammatory change. The score of the semi-quantitative analysis showed a significant increase of severity and extent of inflammatory response in Cd group when compared with NaCl group (Fig 8A and 8B).

Tiotropium inhibited Cd-induced lung inflammatory injuries, with a marked reduction of neutrophil and macrophage infiltration in the alveoli and in the peri-bronchiolar regions and parenchyma. A reduction of focal congestion and hemorrhage was also observed (Fig 7C and 7D). The scores attributed to the inflammatory severity and extent showed a significant reduction in rats pretreated with tiotropium (Fig 8A and 8B).

Pretreatment of budesonide (250 μg/15ml) alone slightly but significantly reduced Cd-induced inflammatory infiltration in the lung tissue (Fig 7E), illustrated by a decrease in mean scores attributed to the severity and the extent of inflammatory injuries (Fig 8C and 8D). A similar protective effect against Cd-induced acute lung injuries was observed in rats pretreated with budesonide and tiotropium (Figs 7F, 8C and 8D), but no interaction between these compounds was noted.

## Discussion

AECOPD is the main cause of high mortality and morbidity of COPD, which is characterized by neutrophil infiltration, airway blockage and sputum production [1]. As one of tobacco components, Cd has been proved to induce acute lung injuries accompanied with an increase of inflammatory mediators and the activity of MMP-2 and MMP-9 in animal models [12,13]. Similar acute inflammatory pathological changes associated with airway hyperresponsiveness were observed in the present rat model. As a toxic heavy metal, Cd has pro-inflammatory properties and can induce the inflammatory responses through several mechanisms [25]. Activation, desquamation and necrosis of epithelial cells may be involved and associated to cytokines and chemokines release [25]. Direct neutrophil activation by Cd has also been reported [26]. Investigation of these mechanisms was out of the scope of this paper. The aim was to explore the pharmacological modulation of tiotropium alone or in combination with budesonide on major inflammatory markers induced by cadmium. Although not been a perfect model of AECOPD, inhalation of Cd in rats can be considered relevant to mimic the main features of AECOPD and be suitable for the research of the mechanism of AECOPD and to investigate the effects of pharmacological agents.

As the first-line treatment of COPD, tiotropium has been reported to effectively improve lung function and quality of life, as well as reduce hospitalization rate by inhibiting bronchoconstriction and mucus secretion [27,28]. *In vivo*, tiotropium has been reported to prevent tracheal hypercontractility in ovalbumin-sensitized guinea pigs [29]. Consistent with these findings, the high concentration of tiotropium was found in the present study to exert a protective effect against Cd-induced airway hyperresponsiveness. Besides the blockage of M3 receptor which gives rise to the bronchodilation, more attention has been paid to the anti-inflammatory effects of muscarinic antagonists [4–6]. In several animal models tiotropium has been found to attenuate acute and chronic pulmonary inflammation [30–32]. Several studies *in vitro* also showed that tiotropium could suppress LPS-induced the release of neutrophil chemotactic mediators by human airway epithelial cells, lung fibroblasts and alveolar macrophages [5,6]. Together with the finding showing inhibitory effect of tiotropium on Cd-induced inflammatory changes in the present model, including a significant decrease of neutrophils and protein concentration in BALF accompanied with a marked attenuation of extent and severity of acute pulmonary inflammatory infiltration, we suggest that the decrease of airway hyperresponsiveness might be related to the anti-inflammatory mechanisms of tiotropium.

Although several mechanisms have been mentioned to explain the anti-inflammatory effects of tiotropium, the findings about the regulation of LAMA on MMP activity in acute lung injuries are quite limited at present. MMP-2 and MMP-9, mainly secreted by a wide variety of cells including alveolar epithelial cells, neutrophils and macrophages [33], are considered as markers of acute lung injuries and play a role in the inflammatory and remodeling process [34–36]. In a cigarette smoke-induced acute lung inflammation mouse model, glycopyrronium has been found to attenuate the elevated MMP-9 expression in lung tissue [22]. Asano *et al*. found that tiotropium inhibited the production of MMP-2 induced by TNF-α or TGF-β in lung fibroblasts [23,24]. The present study showed that the high concentration of tiotropium induced a significant reduction of MMP-2 activity in BALF. However, compared with the pronounced inhibitory effect of tiotropium on neutrophil count in BALF, tiotropium exerted only 10 to 15% inhibition of MMP-2 activity and was quite not effective on increased MMP-9 activity induced by Cd. These findings suggest that other mechanisms are also involved such as the inhibition of chemokine production by inflammatory cells [5,6,37]. While not be significant, the tendency of tiotropium to reduce Il-1β level suggest that this cytokine may be involved in the present model.

Known as a non-specific anti-inflammatory agent, ICSs exert anti-inflammatory effects against asthma and other inflammatory diseases. However, studies showed that ICSs were not so effective in suppressing the inflammatory pathological changes in patients with COPD, indicating that corticoids insensitivity is the main reason affecting the therapeutic efficacy in clinical treatment of COPD [7]. Our previous studies showed that budesonide 500μg/15ml could significantly inhibit Cd-induced acute neutrophilic infiltration in lung tissue associated with a marked reduction of MMP-9 activity in BALF in the present rat model. However, the low concentration of budesonide (250μg/15ml) used in this study had a very limited anti-inflammatory efficacy without any significant impact on MMP-9 activity [38]. Evidence has revealed that LAMA elicited protective effects and reduced the incidence of acute exacerbation in patients with asthma who were resistant to ICS when it was used as an add-on therapy to ICS [39,40]. To observe the regulatory effects of the combination of a LAMA with an ICS on Cd-induced acute neutrophilic pulmonary inflammation, the low concentration of tiotropium (70μg/15ml) was administrated with budesonide (250μg/15ml). These low concentrations were selected in the present study in order to investigate relevant pharmacological concentrations with less additional side effects. Though tiotropium (70μg/15ml) or budesonide (250μg/15ml) could not reduce cadmium-induced bronchial hyper-responsiveness, their combination elicited a significant decrease of E_max._ Additional and synergistic interactions were observed on MMPs activity. Similar result has been reported by the study of Perng *et al*. which showed an attenuation of the increased MMP-9 activity in induced sputum from patients with COPD when an ICS was combined with tiotropium [41]. The combination of both drugs exhibited inhibitory effects on neutrophil count and protein concentration in BALF. Cd-induced increase of severity and extent of lung injuries were also significantly reduced by the combination of both drugs, but no interaction was observed. Surprisingly, the combination of tiotropium with budesonide was slightly less efficient than tiotropium alone on inflammatory cell counts and protein concentration in BALF, but this difference was not significant. When compared the effects of the combination on lung tissue injuries and neutrophil infiltration in BALF, it appears that the impact on histological scores is higher than that on neutrophil count in BALF. This discrepancy could be explained by the fact that histological scores do not only reflect cell infiltration in lungs, but also edema and hemorrhage. On the other hand, recruitment and migration of neutrophils into inflamed lung tissue result in successive different steps, including release, adhesion and migration of neutrophils, modulated by several complex mechanisms differently modulated by pharmacological agents [6, 42–45]. This could explain why the number of neutrophils in BALF and the presence of neutrophils in lung tissue can be differently modulated by tiotropium and budesonide.

## Conclusions

In conclusion, tiotropium exhibited its protective effect against Cd-induced airway hyperresponsiveness and acute neutrophilic inflammatory infiltration. The combination of tiotropium with budesonide inhibits cadmium-induced inflammatory injuries with a synergistic interaction on MMP-2 and MMP-9 activity and airway hyper-responsiveness.

## References

[1] Vogelmeier CF, Criner GJ, Martinez FJ, Anzueto A, Barnes PJ, Bourbeau J, et al Global Strategy for the Diagnosis, Management, and Prevention of Chronic Obstructive Lung Disease 2017 Report: GOLD Executive Summary. Arch Bronconeumol. 2017; 53: 128–149. doi: 10.1016/j.arbres.2017.02.001 28274597 10.1016/j.arbres.2017.02.001

[2] Soler-Cataluña JJ, Martínez-García MA, Román SP, Salcedo E, Navarro M, Ochando R. Severe acute exacerbations and mortality in patients with chronic obstructive pulmonary disease. Thorax 2005; 60: 925–931. doi: 10.1136/thx.2005.040527 16055622 10.1136/thx.2005.040527 PMC1747235

[3] Sapey E, Stockley RA. COPD exacerbations. 2: aetiology. Thorax 2006; 61: 250–258. doi: 10.1136/thx.2005.041822 16517585 10.1136/thx.2005.041822 PMC2080749

[4] Wollin L, Pieper MP. Tiotropium bromide exerts anti-inflammatory activity in a cigarette smoke mouse model of COPD. Pulm Pharmacol Ther. 2010; 23: 345–354. doi: 10.1016/j.pupt.2010.03.008 20362689 10.1016/j.pupt.2010.03.008

[5] Suzaki I, Asano K, Shikama Y, Hamasaki T, Kanei A, Suzaki H. Suppression of IL-8 production from airway cells by tiotropium bromide in vitro. Int J Chron Obstruct Pulmon Dis. 2011; 6: 439–448. doi: 10.2147/COPD.S23695 22003289 10.2147/COPD.S23695 PMC3186742

[6] Vacca G, Randerath WJ, Gillissen A. Inhibition of granulocyte migration by tiotropium bromide. Respir Res. 2011; 12: 24 doi: 10.1186/1465-9921-12-24 21352583 10.1186/1465-9921-12-24 PMC3051905

[7] Barnes PJ. Corticosteroid resistance in patients with asthma and chronic obstructive pulmonary disease. J Allergy Clin Immunol. 2013; 131: 636–645. doi: 10.1016/j.jaci.2012.12.1564 23360759 10.1016/j.jaci.2012.12.1564

[8] Crim C, Calverley PM, Anderson JA, Celli B, Ferguson GT, Jenkins C, et al Pneumonia risk in COPD patients receiving inhaled corticosteroids alone or in combination: TORCH study results. Eur Respir J. 2009; 34: 641–647. doi: 10.1183/09031936.00193908 19443528 10.1183/09031936.00193908

[9] Welte T. Optimising treatment for COPD—new strategies for combination therapy. Int J Clin Pract. 2009; 63: 1136–1149. doi: 10.1111/j.1742-1241.2009.02139.x 19624783 10.1111/j.1742-1241.2009.02139.x PMC2739483

[10] Tumer DI, Ferrari N, Ford WR, Kidd EJ, Nevin B, Paquet L, et al Bronchoprotection in conscious guinea pigs by budesonide and the NO-donating analogue, TPI 1020, alone and combined with tiotropium or formoterol. Br J Pharmacol. 2012; 167: 515–526. doi: 10.1111/j.1476-5381.2012.02016.x 22563753 10.1111/j.1476-5381.2012.02016.x PMC3449257

[11] Kwak MS, Kim E, Jang EJ, Kim HJ, Lee CH. The efficacy and safety of triple inhaled treatment in patients with chronic obstructive pulmonary disease: a systematic review and meta-analysis using Bayesian methods. Int J Chron Obstruct Pulmon Dis. 2015; 10: 2365–2376. doi: 10.2147/COPD.S93191 26604734 10.2147/COPD.S93191 PMC4639518

[12] Blum JL, Rosenblum LK, Grunig G, Beasley MB, Xiong JQ, Zelikoff JT. Short-term inhalation of cadmium oxide nanoparticles alters pulmonary dynamics associated with lung injury, inflammation, and repair in a mouse model. Inhal Toxicol. 2014; 26: 48–58. doi: 10.3109/08958378.2013.851746 24417406 10.3109/08958378.2013.851746 PMC4041479

[13] Bolognin M, Kirschvink N, Leemans J, De Buscher V, Snaps F, Gustin P, et al Characterisation of the acute and reversible airway inflammation induced by cadmium chloride inhalation in healthy dogs and evaluation of the effects of salbutamol and prednisolone. Vet J. 2009; 179: 443–450. doi: 10.1016/j.tvjl.2007.10.004 18037312 10.1016/j.tvjl.2007.10.004

[14] Zhang W, Fievez L, Cheu E, Bureau F, Rong W, Zhang F, et al Anti-inflammatory effects of formoterol and ipratropium bromide against acute cadmium-induced pulmonary inflammation in rats. Eur J Pharmacol. 2010; 628: 171–178. doi: 10.1016/j.ejphar.2009.11.015 19925785 10.1016/j.ejphar.2009.11.015

[15] Pirrone F, Pastore C, Mazzola S, Albertini M. In vivo study of the behaviour of matrix metalloproteinases (MMP-2, MMP-9) in mechanical, hypoxic and septic-induced acute lung injury. Vet Res Commun. 2009; 33 Suppl 1: 121–124. 19578961 10.1007/s11259-009-9255-y

[16] Wang J, Zhang H, Su C, Chen J, Zhu B, Zhang H, et al Dexamethasone ameliorates H2S-induced acute lung injury by alleviating matrix metalloproteinase-2 and -9 expression. PLos ONE. 2014; 9(4): e94701 doi: 10.1371/journal.pone.0094701 24722316 10.1371/journal.pone.0094701 PMC3983216

[17] Kim JH, Suk MH, Yoon DW, Lee SH, Hur GY, Jung KH, et al Inhibition of matrix metalloproteinase-9 prevents neutrophilic inflammation in ventilator-induced lung injury. Am J Physiol Lung Cell Mol Physiol. 2006; 291: L580–587. doi: 10.1152/ajplung.00270.2005 16698855 10.1152/ajplung.00270.2005

[18] Silva PL, Garcia CS, Maronas PA, Cagido VR, Negri EM, Damaceno-Rodrigues NR, et al Early short-term versus prolonged low-dose methylprednisolone therapy in acute lung injury. Eur Respir J. 2009; 33: 634–645. doi: 10.1183/09031936.00052408 19010991 10.1183/09031936.00052408

[19] Wang XQ, Zhou X, Zhou Y, Rong L, Gao L, Xu W. Low-dose dexamethasone alleviates lipopolysaccharide-induced acute lung injury in rats and upregulates pulmonary glucocorticoid receptors. Respirology. 2008; 13: 772–780. doi: 10.1111/j.1440-1843.2008.01344.x 18657064 10.1111/j.1440-1843.2008.01344.x

[20] Mortaz E, Rad MV, Johnson M, Raats D, Nijkamp FP, Folkerts G. Salmeterol with fluticasone enhances the suppression of IL-8 release and increases the translocation of glucocorticoid receptor by human neutrophils stimulated with cigarette smoke. J Mol Med (Berl). 2008; 86: 1045–1056. 18600309 10.1007/s00109-008-0360-0 PMC2517086

[21] Fievez L, Kirschvink N, Zhang WH, Lagente V, Lekeux P, Bureau F, et al Effects of betamethasone on inflammation and emphysema induced by cadmium nebulisation in rats. Eur J Pharmacol. 2009; 606: 210–214. doi: 10.1016/j.ejphar.2009.01.020 19374854 10.1016/j.ejphar.2009.01.020

[22] Shen LL, Liu YN, Shen HJ, Wen C, Jia YL, Dong XW, et al Inhalation of glycopyrronium inhibits cigarette smoke-induced acute lung inflammation in a murine model of COPD. Int Immunopharmacol. 2014; 18: 358–364. doi: 10.1016/j.intimp.2013.12.021 24389380 10.1016/j.intimp.2013.12.021

[23] Asano K, Shikama Y, Shibuya Y, Nakajima H, Kanai K, Yamada N, et al Suppressive activity of tiotropium bromide on matrix metalloproteinase production from lung fibroblasts in vitro. Int J Chron Obstruct Pulmon Dis. 2008; 3: 781–789. 19281093 10.2147/copd.s3945 PMC2650607

[24] Asano K, Shikama Y, Shoji N, Hirano K, Suzaki H, Nakajima H. Tiotropium bromide inhibits TGF-β-induced MMP production from lung fibroblasts by interfering with Smad and MAPK pathways in vitro. Int J Chron Obstruct Pulmon Dis. 2010; 5: 277–286. 20856827 10.2147/copd.s11737 PMC2939683

[25] Olszowski T, Baranowska-Bosiacka I, Gutowska I, Chlubek D. Pro-inflammatory properties of cadmium. Acta Biochim Pol. 2012; 59(4): 475–482. 23240106

[26] Kataranovski M, Mirkov I, Belij S, Nikolic M, Zolotarevski L, Ciric D, et al Lungs: remote inflammatory target of systemic cadmium administration in rats. Environ Toxicol Pharmacol. 2009; 28: 225–231. doi: 10.1016/j.etap.2009.04.008 21784007 10.1016/j.etap.2009.04.008

[27] Salpeter SR. Bronchodilators in COPD: impact of beta-agonists and anticholinergics on severe exacerbations and mortality. Int J Chron Obstruct Pulmon Dis. 2007; 2: 11–18. 18044061 10.2147/copd.2007.2.1.11 PMC2692116

[28] Criner GJ, Bourbeau J, Diekemper RL, Ouellette DR, Goodridge D, Hernandez P, et al Prevention of acute exacerbations of COPD: American College of Chest Physicians and Canadian Thoracic Society Guideline. Chest 2015; 147: 894–942. doi: 10.1378/chest.14-1676 25321320 10.1378/chest.14-1676 PMC4388124

[29] Bos IS, Gosens R, Zuidhof AB, Schaafsma D, Halayko AJ, Meurs H, et al Inhibition of allergen-induced airway remodelling by tiotropium and budesonide: a comparison. Eur Respir J. 2007; 30: 653–661. doi: 10.1183/09031936.00004907 17537779 10.1183/09031936.00004907

[30] Arai N, Kondo M, Izumo T, Tamaoki J, Nagai A. Inhibition of neutrophil elastase-induced goblet cell metaplasia by tiotropium in mice. Eur Respir J. 2010; 35: 1164–71. doi: 10.1183/09031936.00040709 19897560 10.1183/09031936.00040709

[31] Cui Y, Devillier P, Kuang X, Wang H, Zhu L, Xu Z, et al Tiotropium reduction of lung inflammation in a model of chronic gastro-oesophageal reflux. Eur Respir J. 2010; 35: 1370–1376. doi: 10.1183/09031936.00139909 19926736 10.1183/09031936.00139909

[32] Bucher H, Duechs MJ, Tilp C, Jung B, Erb KJ. Tiotropium Attenuates Virus-Induced Pulmonary Inflammation in Cigarette Smoke-Exposed Mice. J Pharmacol Exp Ther. 2016; 357: 606–618. doi: 10.1124/jpet.116.232009 27016458 10.1124/jpet.116.232009 PMC4885512

[33] Goetzl EJ, Banda MJ, Leppert D. Matrix metalloproteinases in immunity. J Immunol. 1996; 156:1–4. 8598448

[34] Coimbra R, Melbostad H, Loomis W, Porcides RD, Wolf P, Tobar M, et al LPS-induced acute lung injury is attenuated by phosphodiesterase inhibition: effects on proinflammatory mediators, metalloproteinases, NF-kappaB, and ICAM-1 expression. J Trauma. 2006; 60: 115–125. doi: 10.1097/01.ta.0000200075.12489.74 16456445 10.1097/01.ta.0000200075.12489.74

[35] Gushima Y, Ichikado K, Suga M, Okamoto T, Iyonaga K, Sato K, et al Expression of matrix metalloproteinases in pigs with hyperoxia-induced acute lung injury. Eur Respir J. 2001; 18: 827–837. 11757634 10.1183/09031936.01.00049201

[36] Pirrone F, Pastore C, Mazzola S, Albertini M. In vivo study of the behaviour of matrix metalloproteinases (MMP-2, MMP-9) in mechanical, hypoxic and septic-induced acute lung injury. Vet Res Commun. 2009; 33 Suppl 1: 121–124. 19578961 10.1007/s11259-009-9255-y

[37] Corry DB, Rishi K, Kanellis J, Kiss A, Song Lz LZ, Xu J, et al Decreased allergic lung inflammatory cell egression and increased susceptibility to asphyxiation in MMP2-deficiency. Nat Immunol. 2002; 3: 347–353. doi: 10.1038/ni773 11887181 10.1038/ni773 PMC2814346

[38] Zhang WH, Zhi JM, Cui YY, Zhang F, Habyarimana A, Cambier C, et al Potentiated interaction between ineffective doses of budesonide and formoterol to control the inhaled cadmium-induced up-regulation of metalloproteinases and acute pulmonary inflammation in rats. Plos One. 2014; 9:e109136 doi: 10.1371/journal.pone.0109136 25313925 10.1371/journal.pone.0109136 PMC4196767

[39] Anderson DE, Kew KM, Boyter AC. Long-acting muscarinic antagonists (LAMA) added to inhaled corticosteroids (ICS) versus the same dose of ICS alone for adults with asthma. Cochrane Database Syst Rev. 2015; 8: CD011397. 10.1002/14651858.CD011397.pub2 PMC8666145 26301488

[40] Matsuse H, Yamagishi T, Kodaka N, Miura A, Kurose Y, Nakano C, et al Tiotropium bromide as add-on therapy to inhaled corticosteroids for treating asthma. Expert Opin Pharmacother. 2015; 16: 1403–1409. doi: 10.1517/14656566.2015.1045877 26001185 10.1517/14656566.2015.1045877

[41] Perng DW, Tao CK, Tsai CC, Liu LY, Lee YC. Anti-inflammatory effects of salmeterol/fluticasone, tiotropium/fluticasone or tiotropium in COPD. *Eur Respir J*. 2009; 33:778–784. doi: 10.1183/09031936.00115308 19129278 10.1183/09031936.00115308

[42] Konrad FM, Braun S, Ngamsri KC, Vollmer I, Reutershan J. Heme oxygenase-1 attenuates acute pulmonary inflammation by decreasing the release of segmented neutrophils from the bone marrow. Am J Physiol Lung Cell Mol Physiol. 2014; 307(9): L707–717. doi: 10.1152/ajplung.00145.2014 25172914 10.1152/ajplung.00145.2014

[43] Zhang Y, Du Z, Zhou Q, Wang Y, Li J. Remifentanil attenuates lipopolysaccharide-induced acute lunginjury by downregulating the NF-κB signaling pathway. Inflammation. 2014; 37(5):1654–60. doi: 10.1007/s10753-014-9893-2 24748477 10.1007/s10753-014-9893-2

[44] Pastemak Y, Yarden-Bilavsky H, Kodman Y, Zoldan M, Tamary H, Ashkenazi S. Inhaled corticosteroids increase blood neutrophil count by decreasing the expression of neutrophil adhesion molecules Mac-1 and L-selectin. Am J Emerg Med. 2016; 34(10): 1977–1981. doi: 10.1016/j.ajem.2016.07.003 27498916 10.1016/j.ajem.2016.07.003

[45] Strandberg K, Bildberg K, Sahlander K, Palmberg L, Larsson K. Effect of formoterol and budesonide on chemokine release, chemokine receptor expression and chemotaxis in human neutrophils. Pulm Pharmacol Ther. 2010; 23(4): 316–323. doi: 10.1016/j.pupt.2010.03.004 20307681 10.1016/j.pupt.2010.03.004

